# Laparoscopic management of small bowel obstruction secondary to a mesodiverticular band of a Meckel’s diverticulum in an adult: A case report and literature review

**DOI:** 10.1097/MD.0000000000039164

**Published:** 2024-07-26

**Authors:** Seito Shimizu, Hitoshi Hara, Yasuhide Muto, Tomoki Kido, Ryohei Miyata, Michio Itabashi

**Affiliations:** a Department of Surgery, Social Welfare Organization Saiseikai Imperial Gift Foundation Inc., Saiseikai Kazo Hospital, Kazo, Saitama, Japan.

**Keywords:** case report, laparoscopic surgery, Meckel’s diverticulum, mesodiverticular band, small bowel obstruction

## Abstract

**Rationale:**

The mesodiverticular band (MDB) of a Meckel’s diverticulum (MD) is a rare, yet notable etiology of small bowel obstruction (SBO) in adults. Due to the nonspecific symptoms and challenging diagnosis thereof, preoperative clinical suspicion and strategic management are crucial for achieving optimal outcomes. Therefore, we presented a case in which laparoscopic surgery was strategically performed to alleviate ileus, due to a preoperative diagnosis of SBO, suspected to be secondary to an MD with a concomitant MDB.

**Patient concerns:**

A 32-year-old male patient presented with a half-day’s duration of epigastric pain, abdominal distension, and tenderness, resulting in the working diagnosis of SBO.

**Diagnoses:**

Initial non-contrast computed tomography (CT) revealed SBO without signs of strangulation, postulated to be caused by an MD and concomitant MDB, resulting in conservative management. The symptoms persisted, necessitating contrast-enhanced CT. However, the dilated bowel loop suggestive of an MD that had been observed on non-contrast CT could not be confirmed on contrast-enhanced CT.

**Interventions:**

Decompression therapy using a long tube provided minimal relief, prompting laparoscopic surgery on the 5th day post-admission for diagnostic and therapeutic purposes.

**Outcomes:**

An MD resection effectively relieved the SBO. The histopathological analysis revealed a true diverticulum with ectopic pancreatic tissue, confirming the diagnosis of an MD. At the band site, vascular and neural structures were encased in a sheath, consistent with the remnants of the vitelline duct mesentery; and histopathologically diagnosed as an MDB. The postoperative course was uneventful, and the patient was discharged on the 9th day, postoperatively.

**Lessons:**

Decompression therapy and strategic laparoscopic surgery based on the preoperative working diagnosis of SBO yielded favorable outcomes, highlighting the importance of the early clinical suspicion of an MD and a concomitant MDB, as the etiology of SBO. The imaging variability and rarity of an MD in adults emphasizes the need for a heightened awareness and an accurate diagnosis for optimal management. Early intervention should be deliberated for patients with suspected intestinal ischemia. However, this case accentuates the clinical implications of strategic planning and employing minimally invasive techniques in the management of an MD-related SBO in adults.

## 1. Introduction

Meckel’s diverticulum (MD), extensively described in 1809, by the German anatomist, Johann Friedrich Meckel the Younger,^[1]^ is the most common congenital gastrointestinal abnormality, affecting 2% to 4% of the population.^[2]^ Arising from an incomplete obliteration of the vitelline duct during embryonic development, an MD is a true diverticulum that frequently contains ectopic gastric or pancreatic tissue.^[3]^

Complications typically occur before the age of 2-years-old,^[4]^ and an MD is generally recognized as an etiology of acute abdominal pain in children. In adults, an MD may be incidentally encountered during surgery for other conditions or discovered due to complications, such as intestinal obstruction, hemorrhage, perforation, and diverticulitis, with a lifetime risk of complications ranging from 4.2% to 9%.^[3,5]^ Treatment typically involves surgical removal of the diverticulum, if the MD is symptomatic-in-nature or causes complications.^[6]^ The mesodiverticular band (MDB), a remnant of the vitelline artery and vein, is commonly found adjacent to the MD. Moreover, approximately 8% of cases of an MD are associated with an MDB.^[4]^

Due to the rarity, nonspecific symptoms, and imaging variability thereof, diagnosing an MD in adults is challenging, frequently resulting in an exploratory laparotomy.^[7]^ Consequently, herein, we presented a case in which laparoscopic surgery was strategically performed to alleviate ileus, due to a preoperative working diagnosis of small bowel obstruction (SBO), confirmed to be secondary to an MD and a concomitant MDB.

## 2. Case presentation

A 32-year-old male patient presented to the emergency department, with epigastric pain of a half-day’s duration. He had no relevant past medical history and was not taking chronic medications. On examination, his vital signs were within the normal range; and the patient was afebrile. Physical examination revealed abdominal distension with softness on palpation. Tenderness was noted throughout the abdomen; however, rebound tenderness was absent, without signs of peritoneal irritation.

**Figure 1.**
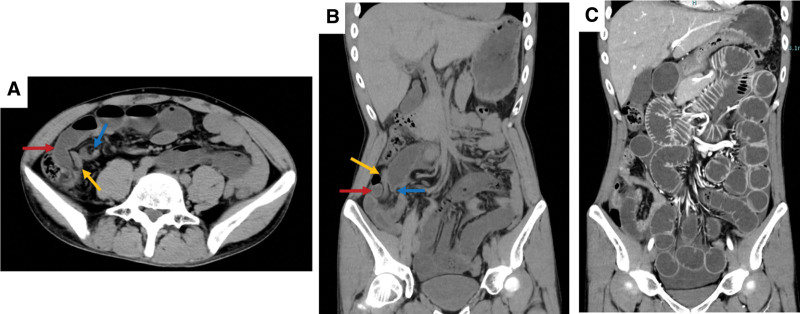
Preoperative computed tomographic (CT) findings are presented. (A and B) Non-contrast CT reveals a narrowing of the terminal ileum (blue arrow), with dilatation of the proximal small bowel. Dilated bowel with a blind end (red arrow) and band-like structure surrounding the small bowel constriction, with a collapse of the distal small bowel (yellow arrow), are shown. These findings are suggestive of an internal hernia, due to a mesodiverticular band of the Meckel’s diverticulum. Signs of strangulation are not evident. (C) Contrast-enhanced CT reveals no evidence of closed-loop obstruction or intestinal ischemia. The dilated bowel loop suggestive of a Meckel’s diverticulum that has been observed on non-contrast CT cannot be confirmed by contrast-enhanced CT.

Non-contrast computed tomography (CT) revealed narrowing of the terminal ileum with dilatation of the proximal small bowel. The working diagnosis of a concomitant MD and an MDB was made, based on the presence of dilated bowel with a blind end, in addition to a band-like structure surrounding the small bowel constriction. Signs of strangulation were not evident (Fig. 1A and B), and conservative treatment with fasting was chosen.

However, little improvement was observed in the symptomatic abdominal distension on the day post-admission. Thus, contrast-enhanced CT was performed. The SBO originating from the terminal ileum remained unchanged on contrast-enhanced CT, with no evidence of closed-loop obstruction or intestinal ischemia. The dilated bowel loop suggestive of an MD that had been observed on non-contrast CT could not be confirmed on contrast-enhanced CT (Fig. 1C).

Differential diagnoses included SBO secondary to a concomitant MD and an MDB, Crohn disease, Behçet disease, and intestinal tuberculosis. Urgent symptoms were not verbalized, nor were findings suggestive of strangulation or ischemia observed.

Decompression therapy was initiated by the insertion of a long tube. Despite tube insertion, the abdominal distension was slightly concerning, and no signs of improvement in SBO were observed. Furthermore, drainage from the tube did not decrease, and an organic stenosis was postulated.

**Figure 2.**
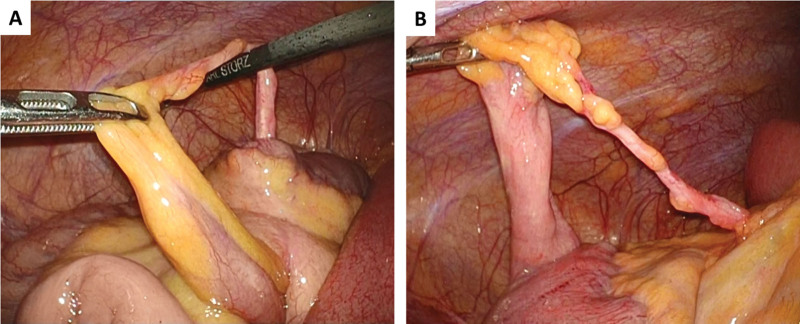
Operative findings are depicted. (A) A segment of the small bowel immediately proximal to the Meckel’s diverticulum (MD) is loosely entrapped, resulting in internal herniation and small bowel obstruction. (B) A mesenteric band continuous with the MD is identified at the base thereof, extending to the band emanating from the tip of the MD, resulting in the diagnosis of a concomitant mesodiverticular band.

Therefore, on the 5th day post-admission, the patient underwent surgery for diagnostic and therapeutic purposes. The surgery was initiated with 3 ports for the laparoscopy: a camera port with a balloon at the umbilicus, and 2 5 mm ports in the left abdomen and middle of the lower abdomen, respectively. Preoperative decompression therapy using a long tube had provided the necessary working space for laparoscopic surgery. The small bowel was diffusely dilated; nonetheless, necrosis was not evident. Additionally, a small amount of serous ascitic fluid was observed. A collapsed small bowel with a blind end was observed in the lower right abdomen, and an MD was identified. A band continuous with the MD was observed, from the tip to near the base thereof, at the point of the mesentery. In the space between them, a segment of the small bowel immediately proximal to the MD was loosely entrapped, resulting in internal herniation and SBO (Fig. 2A). No signs of intestinal ischemia were present. A mesenteric band continuous with the MD was identified at the base thereof, extending to the band emanating from the tip of the MD, resulting in the diagnosis of a concomitant MDB (Fig. 2B).

**Figure 3.**
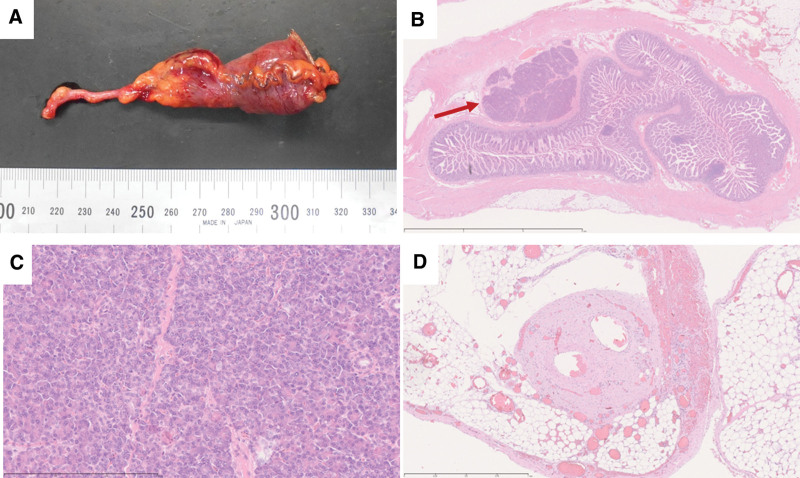
Histopathological findings are depicted. (A) A macroscopic image of the excised specimen is depicted. (B and C) The histopathological analysis of the excised specimen reveals a true diverticulum, involving all layers of the intestinal wall, with ectopic pancreatic tissue (red arrow), confirming the diagnosis of a Meckel’s diverticulum. (D) At the band site, an artery, vein, and neural structures are encased in a sheath, which is consistent with the remnants of the vitelline duct mesentery. Thus, the histopathological diagnosis of a mesodiverticular band has been made.

The entrapped small bowel was gently pulled out, away from the MDB laparoscopically; and the SBO was relieved. The port incision at the umbilicus was extended to approximately 4 cm-in-length; and the small bowel, including the MD, was lifted out of the body. The MD was located 40 cm proximal to the ileocecal valve. The mesentery at the bases of the MD and MDB was ligated and dissected. Thereafter, the MD was resected at the base thereof, using a stapler; and the surgery was completed. The postoperative course was uneventful, and the patient was discharged on the 9th day, postoperatively. The histopathological analysis revealed a true diverticulum with ectopic pancreatic tissue, confirming the diagnosis of an MD (Fig. 3A–C). At the band site, vascular and neural structures were encased in a sheath, consistent with the remnants of the vitelline duct mesentery; and histopathologically diagnosed as an MDB (Fig. 3D).

## 3. Discussion

We encountered a case of a preoperative diagnosis of SBO, postulated to be caused by a concomitant MD and an MDB, in which strategic laparoscopic surgery was performed to relieve the obstruction, post-insertion of a long tube. In this case, MD involvement was postulated preoperatively, due to the identification of MD-like structures and a continuous band-like structure on non-contrast CT, at the timepoint of the initial consultation. However, these structures were difficult to identify on contrast-enhanced CT performed on the day post-admission.

SBO involving the MD is classified into 7 types, as per a study by Amoury et al.^[8]^ As in the present case, an internal hernia caused by an MDB, is classified as type F. However, such cases in clinical practice are rare. Imaging diagnosis is frequently challenging, particularly in adult cases, due to the recognition of an MD as a pediatric condition.^[3]^

In cases of SBO caused by an MDB, such as in this case, the small bowel proximal to the mouth of the MD becomes entrapped in a relatively large gap, mimicking an internal hernia, without considerable dilatation of the MD itself. Notably, the MD is a part of the small bowel; and the appearance thereof on CT imaging may vary depending on peristaltic timing.

In this case, SBO involving the MD was postulated; nonetheless, the presence of a relatively large gap of the loose entrapping of the small intestine due to an MDB, was not evidenced by intestinal ischemia secondary to a closed-loop obstruction. Therefore, decompression was first achieved using a long tube. On the 5th day post-admission, laparoscopic surgery was performed, using a planned strategy. Preoperative decompression facilitated the creation of a working space in the abdominal cavity, allowing for a laparoscopic diagnosis and relief of bowel obstruction with minimal invasion. The MD, which had caused the SBO, could be safely excised through a 4 cm-in-length incision, at the umbilicus.

We reviewed reported cases of SBO caused by an MDB in adults, using the PubMed database. As presented in Table 1, 8 cases have been identified; thus, 9 cases, including the present case, have been assessed.^[7,9–15]^ The average age was 47.4 (26–80)-years-old, with a male-to-female ratio of 2:1. Abdominal pain, nausea, and vomiting were the predominant symptoms. The preoperative diagnosis largely comprised SBO. Including the present case, an MD and MDB were suspected in 4 cases (44.4%) and only 2 cases (22.2%), respectively. The duration from symptom onset to surgery ranged from 1 day to 2 months, with 3 cases (33.3%) requiring emergency surgery on the day of symptom onset. An MD resection was performed in all cases. Small bowel resection was necessary, due to intestinal ischemia in 8 cases (88.8%), apart from the present case in which small bowel resection was avoided. Histopathologically proven MD occurred in 8 cases (88.8%), while histopathological evidence of an MDB was established in only 3 cases (33.3%). Ectopic pancreatic and gastric mucosa were observed in 3 cases (33.3%) and 1 case (11.1%), respectively.

**Table 1:** Summary of reported adult cases of small bowel obstruction caused by mesodiverticular band.

Year	Reference	Age	Sex	Symptoms	Preoperative diagnosis	Time from onset to surgery	Surgical approach	Intestinal resection	Meckel’s diverticulum resection	Pathological findings
2016	Matsumoto et al^[9]^	26	W	Abdominal pain, vomiting	MD, MDB, SBO	2MO	Laparoscopy, mini laparotomy	Yes	Yes	MD, MDB, ectopic gastric mucosa
2017	Yazgan et al^[10]^	35	M	Abdominal pain, nausea, vomiting	MD, strangulated internal hernia	1D	Laparotomy	Yes	NR	NR
2020	Skarpas et al^[11]^	63	W	Distend abdomen, constipation	NR	1D	Laparotomy	Yes	Yes	MD
2020	Ying et al^[12]^	50	M	Abdominal pain, vomiting	SBO	4D	Laparotomy	Yes	Yes	MD
2021	Takura et al^[13]^	56	W	Abdominal pain, vomiting	SBO	1D	Laparoscopy	Yes	Yes	MD, MDB, ectopic pancreas
2022	Alzarea et al^[14]^	57	M	Abdominal pain, vomiting	SBO	3D	Laparoscopy, laparotomy	Yes	Yes	MD, ectopic pancreas
2023	Chaouch et al^[15]^	28	M	Abdominal pain, vomiting	SBO, MD	3D	Laparotomy	Yes	Yes	MD
2023	Zaatar et al^[7]^	80	M	Abdominal pain	SBO	7D	Laparotomy	No	Yes	MD, ectopic pancreas
2024	Our case	32	M	Abdominal pain	SBO, MD, MDB	5D	Laparoscopy, mini laparotomy	No	Yes	MD, MDB, ectopic pancreas

D = day, M = man, MD = Meckel’s diverticulum, MDB = mesodiverticular band, MO = month, NR = not reported, SBO = small bowel obstruction, W = woman.

This case report had several limitations. In this case, no definitive preoperative findings suggestive of intestinal necrosis were observed; therefore, post-decompression, minimally invasive laparoscopic surgery was performed, using an observational approach. However, in cases of SBO involving an MD, intestinal ischemia is not uncommon. In cases of postulated intestinal ischemia, pursuing early emergency surgery is advisable, as opposed to adopting an observational post-decompression approach.

Herein, we described a case in which laparoscopic surgery effectively relieved ileus, preoperatively anticipating SBO due to an MD with a concomitant MDB. An MD with a concomitant MDB can result in rare, yet considerable SBO in adults. This study highlighted that laparoscopic surgery is minimally invasive and provides effective relief with favorable outcomes. Moreover, early consideration of an MD, when the diagnosis of SBO is supported by imaging findings, is essential. Preoperative decompression is a minimally invasive intervention; nevertheless, careful monitoring of intestinal ischemia is of paramount importance. Increased awareness and accurate diagnosis are imperative for optimal management.

## Acknowledgments

The authors thank Dr Hiroko Ogata for the support in the pathological diagnosis and Editage (www.editage.jp) for English language editing.

## Author contributions

**Conceptualization:** Seito Shimizu.

**Investigation:** Seito Shimizu, Hitoshi Hara.

**Project administration:** Hitoshi Hara.

**Resources:** Seito Shimizu, Hitoshi Hara, Yasuhide Muto, Tomoki Kido, Ryohei Miyata.

**Supervision:** Hitoshi Hara, Ryohei Miyata, Michio Itabashi.

**Visualization:** Seito Shimizu, Hitoshi Hara.

**Writing – original draft:** Seito Shimizu.

**Writing – review & editing:** Hitoshi Hara, Michio Itabashi.
